# ASSURED Point-of-Need Food Safety Screening: A Critical Assessment of Portable Food Analyzers

**DOI:** 10.3390/foods10061399

**Published:** 2021-06-17

**Authors:** Safiye Jafari, Julian Guercetti, Ariadni Geballa-Koukoula, Aristeidis S. Tsagkaris, Joost L. D. Nelis, M.-Pilar Marco, J.-Pablo Salvador, Arjen Gerssen, Jana Hajslova, Chris Elliott, Katrina Campbell, Davide Migliorelli, Loïc Burr, Silvia Generelli, Michel W. F. Nielen, Shana J. Sturla

**Affiliations:** 1 Department of Health Sciences and Technology, ETH Zürich, Schmelzbergstrasse 9, 8092 Zürich, Switzerland; jafaris@ethz.ch; 2 CSEM SA, Center Landquart, Bahnhofstrasse 1, 7302 Landquart, Switzerland; davide.migliorelli@csem.ch (D.M.); loic.burr@csem.ch (L.B.); 3 Nanobiotechnology for Diagnostics (Nb4D), Institute for Advanced Chemistry of Catalonia (IQAC) of the Spanish Council for Scientific Research (CSIC), Jordi Girona 18-26, 08034 Barcelona, Spain; Julian.guercetti@iqac.csic.es (J.G.); pilar.marco@cid.csic.es (M.-P.M.); jpablo.salvador@iqac.csic.es (J.-P.S.); 4 CIBER de Bioingeniería, Biomateriales y Nanomedicina (CIBER-BBN), Jordi Girona 18-26, 08034 Barcelona, Spain; 5 Wageningen Food Safety Research, Wageningen University and Research, P.O. Box 230, 6700 AE Wageningen, The Netherlands; ariadni.geballakoukoula@wur.nl (A.G.-K.); arjen.gerssen@wur.nl (A.G.); michel.nielen@wur.nl (M.W.N.F.); 6 Department of Food Analysis and Nutrition, Faculty of Food and Biochemical Technology, University of Chemistry and Technology Prague, Technická 5, Dejvice, 166 28 Prague 6, Czech Republic; Aristeidis.Tsagkaris@vscht.cz (A.S.T.); jana.hajslova@vscht.cz (J.H.); 7 Institute for Global Food Security, School of Biological Sciences, Queen’s University, 19 Chlorine Gardens, Belfast BT9 5DL, UK; J.Nelis@qub.ac.uk (J.L.D.N.); chris.elliott@qub.ac.uk (C.E.); katrina.campbell@qub.ac.uk (K.C.); 8 Laboratory of Organic Chemistry, Wageningen University, Stippeneng 4, 6708 WE Wageningen, The Netherlands

**Keywords:** food safety, portable food analyzer, point-of-need, ASSURED criteria, portable mass spectrometer, optical biosensor, electrochemical biosensor, microfluidic device, lab-on-a-chip, smartphone-based biosensor

## Abstract

Standard methods for chemical food safety testing in official laboratories rely largely on liquid or gas chromatography coupled with mass spectrometry. Although these methods are considered the gold standard for quantitative confirmatory analysis, they require sampling, transferring the samples to a central laboratory to be tested by highly trained personnel, and the use of expensive equipment. Therefore, there is an increasing demand for portable and handheld devices to provide rapid, efficient, and on-site screening of food contaminants. Recent technological advancements in the field include smartphone-based, microfluidic chip-based, and paper-based devices integrated with electrochemical and optical biosensing platforms. Furthermore, the potential application of portable mass spectrometers in food testing might bring the confirmatory analysis from the laboratory to the field in the future. Although such systems open new promising possibilities for portable food testing, few of these devices are commercially available. To understand why barriers remain, portable food analyzers reported in the literature over the last ten years were reviewed. To this end, the analytical performance of these devices and the extent they match the World Health Organization benchmark for diagnostic tests, i.e., the Affordable, Sensitive, Specific, User-friendly, Rapid and Robust, Equipment-free, and Deliverable to end-users (ASSURED) criteria, was evaluated critically. A five-star scoring system was used to assess their potential to be implemented as food safety testing systems. The main findings highlight the need for concentrated efforts towards combining the best features of different technologies, to bridge technological gaps and meet commercialization requirements.

## 1. Introduction

Food safety issues pose serious public health risks worldwide, accounting for 420,000 deaths each year, according to the World Health Organization (WHO) [1]. For instance, a recent food poisoning incident in Uganda in 2019 resulted in 311 illness cases and five fatalities. To identify the cause, the World Food Program halted its Super Cereals aid to many countries suffering from famine. After international investigations, the cause was identified to be Tropane alkaloids, namely atropine and scopolamine contamination by mass spectrometry (MS) and infrared spectroscopy coupled to chemometrics [2]. Considering the complexity of the food chain today, efficient, and reliable food safety systems are crucial to ensure consumer safety and minimize health risks and economic losses. The standard methods for chemical food safety testing rely largely on high-performance liquid chromatography or gas chromatography coupled with MS (LC-MS or GC-MS) [3]. The standard methods for microbiological/pathogen food safety are ISO reference methods based on the culture plate analysis. Although these laboratory-based techniques are highly reliable, they are time-consuming, expensive, and require trained personnel. These requirements limit their wide application across the various stages of complex food supply chains, thus limit the testing frequency and increase contamination risks. Therefore, there is an urgent need to develop portable food analyzers for on-site point-of-need food safety screening. Such tools are expected to ensure consumer safety, particularly in resource-limited settings by allowing for increases in sampling frequency and reducing costs. 

Recently, biosensors have gained increasing attention as screening methods for on-site food safety testing [4,5]. Biosensors are often integrated into small bioanalytical devices, which provide rapid, selective, and sensitive detection of analytes in samples. They consist of three main components:

- The biorecognition element, in general, an antibody, aptamer, or enzyme that binds specifically to the target analyte;
- The transducer converts resulting signals, which can be optical, electrochemical, magnetic, calorimetric, etc.;
- The readout system is used to visualizes the result.

Different types of biosensors have been combined with either paper-based or chip-based microfluidics to form lab-on-a-chip devices, which provide a powerful tool for point-of-need food testing. Although these devices provide rapid and user-friendly analysis, based on performance criteria set out in EU regulations (EC) No 882/2004 and (EU) No 519/2014, they are considered screening tests. As screening methods, they are meant to differentiate between large numbers of compliant (negative) samples and a few suspects (positive) samples. According to EU regulation 2002/657/EC, suspect results require follow-up by a confirmatory instrumental method to declare those samples either compliant or non-compliant [6]. In this regard, recent advancements in portable mass spectrometers could eventually enable confirmatory MS analysis to be performed on-site [7]. As the nearest technology to the gold standard, we decided to investigate the status of portable MS technology in the food safety field to have a comprehensive picture of the portable food analyzers. While the performance of portable MS could already meet screening analysis requirements, instruments cost between 100,000–300,000 euros which is more than 1000 times higher than that of biosensing devices. Therefore, a clear goal should be to achieve performance specifications sufficient to qualify the portable mass spectrometer for on-site confirmatory analysis. 

In this review, we aim to provide key insights into the technological advancements of miniaturized and integrated food analyzers based on state-of-the-art electrochemical and optical biosensing platforms. The ASSURED criteria correspond to Affordable, Sensitive, Specific, User-friendly, Rapid and Robust, Equipment-free, and Deliverable to end-users. These criteria were set by the WHO for point-of-care diagnostic test performance evaluation in resource-limited settings and are complementary to the performance criteria for screening and confirmatory methods in the EU regulation 2002/657/EC [6,8]. Therefore, we considered them as the basis of comparing the performance of portable food safety analyzers in this review article. Affordability is considered as the cost of the test and the additional equipment necessary for realizing the test. In the ASSURED criteria, Sensitivity is defined as true positive rate and Specificity as true negative rate. However, data on true positive and negative rates, for the examples evaluated for this study, were often not provided with validation studies based on the regulatory guidelines for such an assessment (EU regulation (EU) No 519/2014). Therefore, we chose to consider the limit of detection (LOD) compared to the maximum residue limit (MRL) indicated in EU regulations as a proxy for Sensitivity, and the selectivity (the analyte signal relative to interferences of similar molecules or other contaminants) as a proxy for Specificity [6]. User-friendliness is evaluated by simplicity, automation, and minimum training required for performing the test. To complete the evaluation, we addressed Rapidness as total analysis time, Robustness as test vulnerability toward minor changes in assay conditions, Equipment-free as the need for extra equipment, and Deliverable to end-users as the accessibility of the technology to the public. 

For each of these terms, a five-star grading was used to compare different classes of portable devices as shown in Table 1. The grading was in part subjective to our assessment of the reviewed papers in this work to reflect the status of portable food safety analyzers. The assessed overall score was calculated as the average of stars for all the terms in the ASSURED criteria. Based on our grading, paper-based colorimetric and smartphone-based devices are the most promising technologies for on-site food analysis. This ranking is following the trend observed in devices already on the market, namely paper-based test strips. An in-depth discussion of the scoring of each of the technologies in Table 1 is reported in the following sections. Finally, the potential role of portable MS analyzers for food analysis has been evaluated and the challenges of bringing the confirmatory MS technique from the laboratory to the field are discussed. While biosensing devices as screening tests are complementary to confirmatory methods (LC- or GC-MS), portable MS devices could potentially replace both methods.

## 2. Portable Optical Food Analyzers

Optical biosensing platforms are based on measuring changes in properties of light, such as intensity, wavelength, polarization, or propagation direction as the biosensor response. These changes can be monitored using different methods, such as colorimetric, fluorescence, surface plasmon resonance, infrared, and Raman spectroscopy [9]. The integration of these optical techniques with paper-based, microfluidic chip-based, and smartphone-based platforms has been considered for on-site food analysis. Optical transducers are versatile, spanning read-outs ranging from those distinguished by the naked eye in colorimetric detection to involving spectrometers and refractometers. Colorimetric detection with the naked eye is the cheapest biosensing platform, however, it can only provide qualitative analysis. Using an imaging system such as a reader or a smartphone improves the sensitivity to a semi-quantitative level but introduces more cost and decreases user-friendliness. On the other hand, optical detection based on spectrometry and refractometry, which provides quantitative analysis, is usually performed using lab-based benchtop UV-Vis and fluorescence spectrometers [10]. Recently, there has been a trend toward the development of miniaturized spectrometers with a similar performance to benchtop devices at a lower cost. While this goal is particularly challenging, considering the fabrication of miniaturized gratings and reflective optics, and decreasing the path length, some of these devices are already available on the market at a few thousand dollars [11]. 

### 2.1. Paper-Based Optical Food Analyzers

Paper-based optical food analyzers with simple colorimetric detection are the most common type of portable food analyzers (Table S1). Simple paper-based assays as test strips are commercially available for various food contaminants such as aflatoxins, marine biotoxins, and pesticides. The two main categories of these devices are lateral flow assays (LFA) and microfluidic paper-based analytical devices (µPADs), both providing rapid, low-cost, and user-friendly on-site analysis [12,13]. While the sample flow in LFA only moves in one direction, microfluidic channels in µPADs can direct the flow in complex patterns enabling multi-step procedures [14].

From the ASSURED criteria, these devices meet ideally the affordability (five stars), specificity (four stars), and rapidness (five stars) criteria. Also, they are equipment-free (five stars) and commercially available, thus immediately and easily deliverable to end-users (five stars). However, they only receive one star in sensitivity since the results are qualitative or semi-quantitative due to the intrinsic limitations of the technology. Their overall score is 4.3 (Table 1), top-ranked amongst the portable food analyzers when all criteria are weighted equally. 

One of the main reasons for the popularity of paper-based devices is the paper itself. The cellulose membrane not only provides an immobilization platform for biorecognition elements, such as antibodies or aptamers but also acts as a transportation and reaction platform for the sample and other reagents. The LFA mainly uses nitrocellulose as the support material, owing to its excellent protein adsorption properties for the immobilization of biomolecules. Their rapidness was demonstrated in a study combining LFA with nucleic acid extraction, amplification, and colorimetric detection of *Escherichia coli* and *Streptococcus pneumonia* in milk and spinach [15]. Compared to time-consuming conventional culture plate assays, which require more than five hours, this hybrid LFA provided the results within one hour. Another rapid LFA example was developed for the detection of hazelnut allergens in cookies with a carbon nanoparticle-labeled antibody. The qualitative result in this example was displayed in 30 s [16]. 

**Figure 1:**
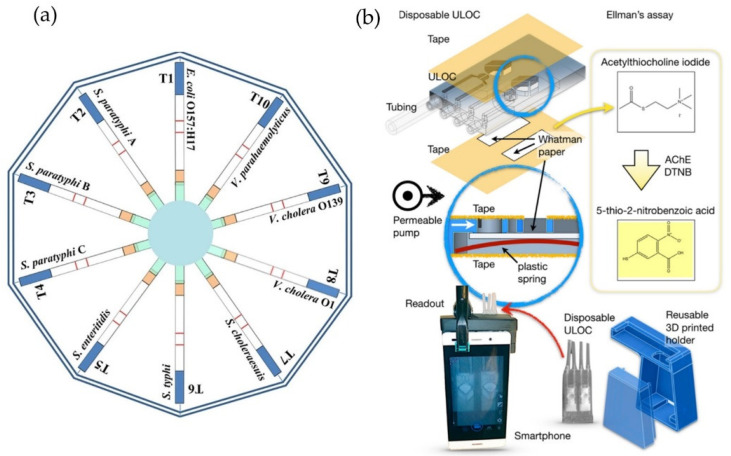
Examples of hybrid paper-based optical food analyzers: (**a**) a disc containing 10 different LFA assays for foodborne pathogen detection. Reproduced with permission from [18] Scientific reports, Copyright (2016), under CC BY 4.0; (**b**) a hybrid paper-LOC device provides semiquantitative carbofuran screening using a smartphone as a detector. Reproduced with permission from [19] Sensors, Copyright (2019), under CC BY 4.0.

Multiplexing can further increase the performance of paper-based assays and bring it closer to the traditional microplate assays with high-throughput analysis capability [17]. In this regard, rapid multiplex detection of 10 different foodborne pathogens in different food matrices (dairy products, marine products, beverages, snacks, and meats) was achieved within 20 min using a disc with multiple paper-based LFA devices [18]. The geometry of this hybrid assay permits simultaneous sample injection into all the LFAs, making it simple and user-friendly (Figure 1a). Another example is a hybrid paper-lab-on-a-chip (paper-LOC) injector for carbofuran screening in apple extracts [19]. The paper-LOC device is a low-cost and multiplexed device, with a price of 0.30 euro for analyzing two samples. However, the device has low sensitivity with an LOD of 0.050 mg∙kg^−1^, which is 50 times higher than the MRL for carbofuran (0.001 mg∙kg^−1^) set by the EU commission regulation (part A of Annex I to Reg. 396/2005). This device also features integrated sample handling, with sample and reagent injection using silicone tubing (Figure 1b). 

Although colorimetric detection with the naked eye is a simple approach with a high level of user-friendliness, enabling non-experts to perform on-site food analysis, measurements are not always accurate and/or sensitive enough. Furthermore, the colorimetric signal can easily be affected by (ambient) light conditions, color metamerism, and colored food matrices set another challenge for creating a successful color-based detection system [20]. To circumvent such limitations, paper-based assays have been combined with various spectroscopic detection systems. Chemiluminescent detection was utilized to detect dichlorvos, an organophosphate insecticide in vegetables, in a wide linear range from 0.006–2 mg·kg^−1^ and with an LOD equal to 0.0016 mg∙kg^−1^, which is lower than the regulatory limit of 0.01 mg∙kg^−1^ [21]. Localized surface plasmon resonance was also used for biogenic amine detection for monitoring salmon freshness. In this case, nanoparticle-embedded papers served as gas sensors, providing high particle transfer efficiency and a strong resonance reflectance dip [22]. While these examples showcase the potential for integration of spectroscopic and paper-based systems, they increase the cost and reduce the user-friendliness of paper-based devices.

### 2.2. Microfluidic, Chip-Based Optical Food Analyzers 

Microfluidics systems provide precise control and manipulation of small amounts of fluids using microchannels [23]. The microchannels are fabricated mainly using transparent polymers such as polydimethylsiloxane (PDMS), and poly(2,5-dimethoxyaniline) (PDMA), adhered to a glass substrate [24]. This relatively new field became more popular with the development of 3D printing technologies providing easy and cost-effective manufacturing of the prototypes. The microfluidic devices can mimic reactors to carry out sample preparation, filtration, dilutions, and detection, which results in a reduction of handling errors and a subsequent increase in analytical robustness [25]. These advantages are of great importance in the development of an integrated portable food analyzer (Table S2). Considering the ASSURED criteria, microfluidic chip-based optical food analyzers provide sensitive and specific analysis with four stars for each of these parameters. However, they require additional equipment, such as a built-in camera, optical filters, illumination sources, and pumps, which reduces affordability (two stars) and limits accessibility (two stars). Compared to other devices, they obtain three stars in rapid and robust and two stars in user-friendly terms, resulting in an overall score of 2.6. The liquid handling automation, the potential for high-throughput, and integration of sample preparation in these devices might increase their appeal for on-site analysis. 

**Figure 2:**
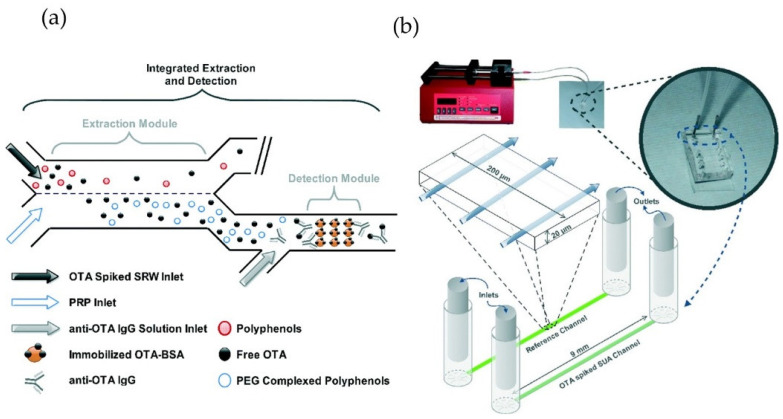
An example of a microfluidic chip-based optical food analyzer used for ochratoxin A (OTA) detection in red wine. (**a**) The integrated microfluidic strategy for performing an aqueous two-phase extraction with PEG-rich phase (PRP); (**b**) After performing an indirect competitive fluorescence-linked immunosorbent assay, the measurements are performed with a fluorescence microscope under continuous flow. Reproduced with permission from [26] Lab on a Chip, Copyright (2014), under Royal Society of Chemistry.

In general, optical biosensing is a multi-step procedure, involving extraction of contaminants from the food sample, transport to the biorecognition element, and optical transduction. Integrating these steps into a single microfluidic chip-based device can effectively improve the applicability of optical food analyzers in real-life settings. Toward this goal, an integrated microfluidic device was fabricated for simultaneous extraction, preconcentration, and detection of ochratoxin A in wine. The analysis time was less than 30 min with an LOD of 0.26 µg∙kg^−1^, which is 8 times lower than the EU regulatory limit of 2 µg∙kg^−1^ (Figure 2) [26]. The PDMS-based device consisted of two consecutive modules performing a two-phase extraction and immunoassay detection, but a microscope was needed for quantitative analysis. The authors suggest replacing the microscope by integrating the device with an on-site fluorescence photodetector. Moreover, the measurement was performed under continuous flow using syringe pumps, which provides automation but reduces user-friendliness for non-expert users. 

Many lab-on-a-chip platforms in the food safety field are designed for the detection of pathogens, which requires integrating the sample preparation, isothermal amplification, and detection processes in a single device. The reported studies combine nucleic acid recognition, polymerase amplification, and fluorescence imaging to detect bacteria like *Salmonella typhimurium* and *Escherichia coli* with limits of detection of 4 to 10 cells·µL^−1^ in milk and cultured media [27,28]. These devices mostly use centrifugal platforms in the shape of a compact disc, consisting of different chambers for extraction, amplification, and detection steps. The fluid flow between chambers, the reaction, and the mixing procedures are controlled by centrifugal force, eliminating the need for pumps, valves, and tubes. Although these devices provide sensitive analysis below the MRL set by EU regulations, they require a centrifuge and a miniaturized spectrometer. To improve the sensitivity even further, one outstanding study reported integrating a supercritical angle fluorescence microlens array in a microchip. The microlens has a high fluorescence collection efficiency, which resulted in higher sensitivity and LOD of 1.6 copies·µL^−1^ of pathogenic DNA. The result was comparable to the conventional spectrometers’ performance but with a higher noise level and a lower signal to noise ratio [27]. 

Other optical detection methods, such as surface plasmon resonance (SPR) were also employed for optical microfluidic chip-based food analysis. While SPR provides label-free optical detection, it has a lower sensitivity compared to fluorescence-based detection, particularly for the detection of small molecules, such as mycotoxins due to the low refractive index change. In some studies, the SPR sensitivity was improved by signal amplification using nanomaterials. An integrated gold chip with gold nanoparticles was reported for the detection of aflatoxin B1 in wheat with an LOD of 0.094 µg·kg^−1^, which is 20 times lower than the EU regulatory limit of 2 µg∙kg^−1^ [29]. This device used gold nanoparticles to amplify the SPR signal, thus improving the sensitivity. Another study implemented a 3D dextran layer on a nanostructured imaging surface plasmon resonance chip for signal amplification. This device was used for the detection of deoxynivalenol and ochratoxin A in beer with LODs of 17 ng∙mL^−1^ and 7 ng∙mL^−1^, respectively. Interestingly, the study reported a preliminary in-house validation with 20 beer samples. While this device was able to detect deoxynivalenol below regulatory limits for beer, pre-concentration of the sample was required before detection of ochratoxin A [30]. The chip could be reused 450 times, which reduces the cost of each test. Another analogous strategy for reducing cost was reported involving a regenerable glass biochip for detection of ochratoxin A in coffee beans, reusable up to 20 times [31]. With this chemiluminescence-based device, researchers were able to detect ochratoxin A with an LOD of 7 µg·kg^−1^ and a total analysis time of 12 min. However, the use of an immunoaffinity column for sample enrichment introduced more cost and decreased user-friendliness. In terms of the total analysis time, most of the integrated devices provide the results in less than one hour, including sample preparation [32,33,34,35].

### 2.3. Smartphone-Based Optical Food Analyzers 

Integrating optical detection into smartphone-based biosensors takes advantage of the smartphone optical components, such as the light source and the camera as a photodetector. The crucial aspect here is that a smartphone not only detects but also provides the location (GPS), time, and wireless data transfer to stakeholders, thus enabling the geo-temporal mapping of (food) contamination issues. From the ASSURED criteria, these devices improve user-friendliness and accessibility of on-site food testing (4 stars). When compared to other portable food analyzers, they were rated with four stars in terms of affordability, user-friendliness, equipment-free, and deliverable to end-users. They have a slightly better sensitivity (two stars), compared to equipment-free paper-based optical devices. Their overall score, therefore, is 3.4, which places them amongst the top three most promising portable food analyzers. 

Smartphone-based optical food analyzers are mainly used with colorimetric detection, which results in a picture to be analyzed using a smartphone app based on RGB, CieLAB, HSV, or greyscale. The functioning of these color spaces for optimal sensitivity and error reduction has been investigated extensively. It was found that individual RGB channels often produce optimal colorimetric performance, although other color spaces such as CieLAB or even novel artificial channel combinations of various color spaces combined may equally perform very well in certain assays [36]. The reviewed smartphone-based colorimetric detection strategies are based on the immunoassay test strips [37,38], nanoparticle aggregation [39,40], or enzyme inhibition assays [41,42]. Although the limits of detection of the colorimetric smartphone-based food analyzers reported in the literature are fit for screening methods based on the food safety regulations, they only provide qualitative or semi-quantitative results. 

Alternative approaches such as fluorescence and chemiluminescence have been explored to provide quantitative results for the detection of mycotoxins in corn and tetracyclines and quinolones in milk, respectively [37,43]. With these methods, pesticide detection in spinach with LOD 5 to 10 times lower than the regulatory limit was achieved, affirming them as fit for purpose [44]. Furthermore, the fluorescence method is preferred over chemiluminescence for pathogen detection since fluorescence imaging directly detects the fluorescent-labeled molecules and does not require an enzymatic reaction to produce the detection signal. Smartphone-based fluorescence assays were reported with detection limits of 58 colony-forming unit (CFU)·mL^−1^ in fruit juice and 1 CFU·mL^−1^ in yogurt for *Salmonella typhimurium*, and 10 CFU·mL^−1^ for *Escherichia coli* in egg samples, which were all lower than the EU regulation limits [45,46].

**Figure 3:**
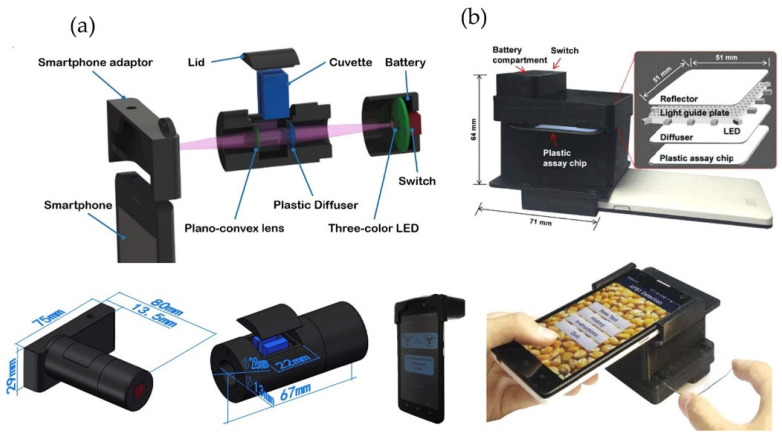
Examples of smartphone-based optical food analyzers: (**a**) The 3D printed smartphone optosensing platform for detection of streptomycin in honey and milk. The anti-streptomycin aptamer-conjugated gold nanoparticles are used as the colorimetric indicator. The ratio of the absorbance at 625 nm to that at 520 nm was measured as the optosensor signal. Schematic overview of the internal structure and dimensions. Reproduced from [47] Analytica Chimica Acta Copyright (2017), with permission from Elsevier; (**b**) The integrated Smartphone-App-Chip system for aflatoxin B1 detection in corn. The detection is based on an indirect competitive immunoassay in the microfluidic channel. Then the assay chip is aligned in the 3D-printed optical accessory attached to a smartphone. The image captured by the smartphone camera is directly processed using a custom-developed Android app. Reproduced with permission from [48] Analytical Chemistry, Copy right (2017), American Chemical Society.

All the assessed optical smartphone-based devices required external equipment and accessories. Mainly 3D printed modules are produced to place the smartphone in a fixed position and acquire the data in a reproducible manner. This could reduce the effect of external variations, like illumination conditions and user handling errors, while improving the sensitivity. An optosensing 3D printed platform was developed for quantitative colorimetric detection on the smartphone (Figure 3a). This device was validated with a UV-Vis spectrometer for the detection of streptomycin in honey and milk with an LOD of 0.009 mg·kg^−1^ [47]. The anti-streptomycin aptamer-conjugated gold nanoparticles were used as the colorimetric indicator. The ratio of the absorbance at 625 nm to that at 520 nm was measured as the optosensor signal. One of the main advantages of 3D printing is the possibility of realizing rapid prototyping of disposable microfluidic chips at a low cost. A few papers reported estimations of the final cost of the device. For example, the above-mentioned article reported a price of 13 US dollars per device and 5 US dollars per test [47]. Another study reported a total cost of 5.20 US dollars for colorimetric detection of aflatoxin B1 in corn samples (Figure 3b) [48]. The detection was based on an indirect competitive immunoassay in the PDMS microfluidic channel. Then, the chip was aligned in the 3D-printed optical accessory attached to a smartphone. The image captured by the smartphone camera was directly processed using a custom-developed Android app. The reported price is comparable to the 96 microwell plate Enzyme-linked Immunosorbent Assay (ELISA) or 3–10 US dollars per sample. These numbers suggest a high commercial potential for smartphone-based optical food analyzers. Since the inter-phone variability has proven to be a major hurdle for the implementation of these devices, the evaluation of different smartphone brands has been reported in several studies. The device-independent color space and randomized combined channel approaches were used for smartphone-based image analysis to reduce the interphone variations [36,49].

The food matrix also has a defining role in the development of a smartphone-based optical food analyzer. Liquid matrices such as milk, different types of beverages like juices and cider are the preferred matrices, as they do not require complex extraction procedures (see Table S3). This is mainly due to the complexity of integrating sample preparation with a detection platform in a single device, which limits the applicability of the developed food analyzers. In general terms, it has been observed that further effort in development is needed to simplify the complex extraction protocols for non-expert users. 

Even though the time of the procedure varies in most of the cases analyzed, the measurement time ranges between five minutes in the best cases and up to 45 min [37,44]. Extra time may be required for sample preparation depending on the composition of the matrix and can take from 10–45 min. 

### 2.4. Raman and IR-Based Portable Food Analyzers

Although vibrational spectroscopy, infrared (near-IR and mid-IR), and Raman spectroscopy, has been mostly applied to reveal food adulteration or verify the origin and authenticity of food, there is a trend towards its implementation in the food safety field (Table S4) [50,51]. Considering the ASSURED criteria, these devices are rapid and robust (four stars) and provide high analytical specificity (five stars). They are relatively user-friendly (three stars) as the analyses are non-invasive and require minimal sample preparation. This feature is a big advantage when compared to multi-step sample preparation required for other devices, from grinding the solid sample and using organic solvent for extraction, to filtration and dilution. Moreover, portable IR and Raman spectrometers have recently become widely available, further increasing the applicability of such platforms in bringing food analysis to the field [52]. While a handheld NIR device such as SCiO is commercially available for only 600 euros, portable Raman spectrometers are generally available at a cost of few thousand euros [53]. Therefore, they are assigned only two stars in affordability and equipment-free terms. Also, they suffer in terms of sensitivity (one star), as the quantitative analysis is still a challenge. Their overall score is 2.6. 

Surface-Enhanced Raman Spectroscopy (SERS) can either directly screen for an analyte or can be complementary to other screening methods such as bioanalytical assays to enhance specificity and sensitivity. Signal enhancement of the Raman scattering is necessary for a range of applications and can be achieved using different colloidal or solid nanocomposite substrates [54]. The solid substrates can be immobilized on various surfaces, for example, paper or hydrogels [55,56]. The paper-based SERS substrates can be used at the same time as a sample collection tool to swab the surface of a sample. Although combining a bioanalytical method with SERS can drastically reduce the attained LOD, it increases the method’s complexity and cost. For example, coupling a lateral flow immunoassay to SERS resulted in 3 orders of magnitude lower LOD (reaching the pg· mL^−1^ level) compared to the colorimetric naked-eye detection for the antibiotics neomycin and quinolone in milk [57].

It is important to underline that acquiring quantitative results using SERS remains a challenge, even though more and more studies report their results as quantitative (see Table S4). The use of anisotropic nanoparticles (nano-cubes, nano-rods, and nano-stars) and internal standards has positively affected SERS quantification capabilities [58]. In the study reported by X. Li et al., water molecules were used as the internal standard, since their Raman scattering signal was quite stable [59]. In another study, 4-methylthiobenzoic acid (4-MBA), a Raman active probe molecule, was embedded in gold-silver core-shell nano-cubes and exploited as the internal standard to attain quantitative results [60].

**Figure 4:**
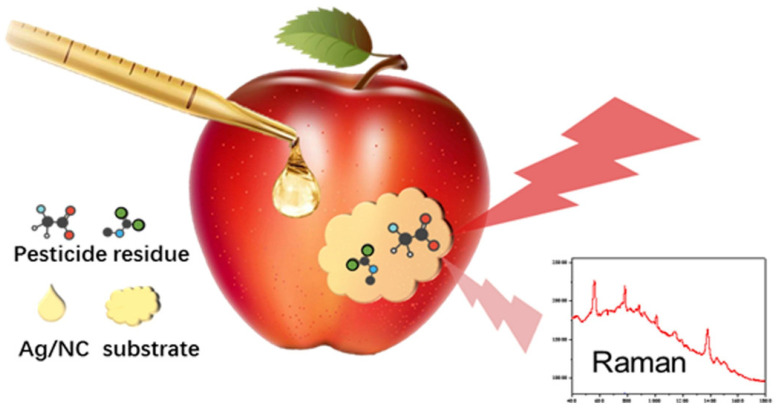
SERS application for rapid in-situ pesticide detections in fruits and vegetables. The nanocellulose decorated with Ag nanoparticles (Ag/NC) substrate was used to detect pesticides (thiram and thiabendazole) on apple and cabbages samples. Reproduced from [62] Carbohydrate Polymers, Copyright (2019), with permission from Elsevier.

Another challenge is that SERS can mostly detect analytes on the surface of food, which does not correspond to the whole amount of a contaminant in a food matrix. One example is pesticide residues, which can be found on the surface or in the inner parts of fruit, depending on their polarity. In such cases, the LOD is in general expressed using the unit “ng·cm^−2^”, which is not in line with the MRL units (mg∙kg^−1^) (Figure 4) [61,62]. In some other studies, an extraction method was used before SERS screening, an approach that eliminates one of the most desirable characteristics of SERS: being a non-invasive method with no sample preparation [63].

Near-IR (NIR) spectroscopy is another method that has been applied for food contaminant screening with minimum sample preparation. The typical NIR spectrum consists of complex, broad, and superimposed absorption bands of low intensity. Consequently, NIR provides mostly qualitative results and the application of chemometric tools is necessary to achieve an effective screening. Among various chemometric models, partial least square regression and linear discriminant analysis have been applied for the screening of both pesticides and mycotoxins [64,65]. These models are based on a linear relationship between the NIR spectrum intensity and concentration. They use multi-variable data analysis (multiple wavelengths) with as many as 20–1000 variables to achieve quantification. 

The mid-IR spectrum consists of strong vibrational bands in the molecular fingerprint region. The mid-IR spectral intensity is a thousand-fold higher than in the NIR, thus achieving higher sensitivity of the measurements. However, the mid-IR is limited to solid food samples with low moisture content, due to the strong water absorption in this wavelength range. Fourier transform infrared (FT-IR) spectroscopy in mid-IR was reported for the identification of different pathogenic bacteria [66]. The data was obtained using hyperspectral imaging and the data analysis was performed by image segmentation with machine learning and artificial neural networks. The sample preparation included growth and cultivation in an agar plate and subsequent dilution and drying of the sample on an imaging substrate. Another study reported *Botrytis cinerea* fungus detection on tomato leaves in both visually symptomatic and pre-symptomatic plants [67]. The detection accuracy was 100% using chemometrics data analysis at different stages of the disease with a semi-portable spectrometer. A total number of 240 spectra was acquired from 16 leaf samples for accuracy studies. The high detection accuracy for both true-positive and true-negative rates of 100% was also reported for the detection of aflatoxin B1 and total aflatoxins in 90 peanut oil samples. Samples with the concentration higher than 20 µg∙kg^−1^ were identified as positive for both aflatoxin B1 and total aflatoxins detection. This threshold is higher than the EU MRLs for both aflatoxin B1 and total aflatoxins as 2 µg∙kg^−1^ and 4 µg∙kg^−1^, respectively [68]. Quantitative detection was achieved using FT-IR for the detection of clenbuterol in beef meat with an LOD of 2 µg·kg^−1^, which is 20 times higher than the regulatory limit of 0.1 µg∙kg^−1^ (Commission Regulation (EC) No 1312/96) [69]. Despite a 100% accuracy, the number of samples was limited, and the sensitivity of these studies was not fit for the purpose concerning regulatory requirements.

## 3. Portable Electrochemical Food Analyzers

Electrochemical biosensors have a great potential in portable analysis owing to the ease of miniaturization and integration of the transducer while providing quantitative results. The electrochemical transducer consists of an electrochemical cell connected to a potentiostat/galvanostat, which performs the measurements. The traditional electrochemical setup is a benchtop device with the standard electrodes in millimeter dimensions. However, the electrochemical cell has been miniaturized at low cost with screen printing or inject printing of the 3 electrodes, reducing the required sample volumes to few microliters [70,71]. Apart from the commercially available miniaturized potentiostats, there has been an increasing trend towards the development of open source and cost-effective portable potentiostats for resource-limited applications [72,73]. The open-source potentiostat equipped with wireless connectivity to the smartphone developed by Whiteside’s group is a recent example of such an instrument [74]. The total cost of a single device was 61.5 US dollars, which can be reduced to 15 US dollars if produced in large numbers. This is considerably cheaper compared to the cost of commercial laboratory potentiostats, which typically range in a few thousand dollars. 

No matter which technique is used, the key feature of electrochemical detection is providing intrinsic quantitative results [75]. Moreover, the electrochemical signal is not affected by ambient illumination conditions and the color or turbidity of the sample. This is an extra advantage in the food safety field, where extracts often remain colored or exhibit a certain level of turbidity, which can interfere with optical measurements [20,76]. However, electrochemical detection can be affected by electrode surface fouling and/or poisoning caused by food matrix components, affecting the robustness of measurements. The fouling issue can be circumvented using magnetic beads to extract the analyte from the matrix. This technique was successfully applied to detect domoic acid in shellfish below MRL. The cost-effective nanomaterial carbon black (approximately one US dollar per kg) was used to reduce background noise and improve sensitivity [77,78].

Alongside the miniaturization of the sensing platform, other procedures such as extraction, separation, and washing steps should be miniaturized and automated to bring the laboratory-based food safety tests to the field. The electrochemical biosensor’s ease of miniaturization and integration makes them a suitable choice to be incorporated in either paper-based or chip- microfluidic devices, designed for automation and miniaturization of the sample extraction and handling processes [79]. Moreover, the potentiostat connectivity to the smartphone is an excellent way to make the electrochemical food safety analysis more user-friendly, minimizing the need for user training [80]. Here, examples of portable electrochemical biosensors in the food safety field are categorized in terms of their integration formats into paper-based, chip-based, and smartphone-based microfluidic devices.

### 3.1. Paper-Based Electrochemical Food Analyzers

Integrating an electrochemical biosensor into a paper-based device can provide more sensitive and quantitative results compared to colorimetric equivalents (lateral flow assay and µPAD) [81]. The microfluidic paper-based electrochemical device, or µPED, was introduced quickly after the µPAD both by Henry’s and Whiteside’s groups independently in 2009 and 2010 [82,83]. Since then, the µPEDs have been developed mainly as diagnostic tests, while their application in the food safety field remains quite limited, with only seven studies addressing the detection capabilities of the developed µPED devices in food matrices (Table S5) [84]. Despite being quite new, this integration strategy is particularly promising for the food safety field, with its potential to combine affordability of paper-based assays and sensitivity of the electrochemical analysis. Although the integrated device provides improved sensitivity, ranked two stars over the optical paper-based devices with only one star, this comes at the expense of affordability, being equipment-free and deliverable to end-user terms, due to the need for a potentiostat and electrodes. The electrochemical devices are also less user-friendly compared to optical paper-based devices with only three stars. Thus, their overall score is 2.6. 

As affordability and user-friendliness are the key features of colorimetric paper-based devices, the competitiveness of the µPED depends directly on the development of affordable, user-friendly portable potentiostats and low-cost electrodes. Although there are some good examples of low-cost portable potentiostats in the literature, they still lack true user-friendly interfaces and are not comparable to colorimetric assays. However, the electrodes could be integrated into the paper substrate at a very low cost using different techniques, such as screen-printing, inkjet printing, and microwires [85]. The study performed by Araujo et al. is a good example of minimizing the cost of carbon electrode integration in the paper through laser scribing pyrolysis with a CO_2_ laser [86]. This method is not only low cost at 0.025 US dollars per device, but also environmentally sustainable, not requiring a multi-step printing process using conductive inks and other chemicals, as the screen-printing technique does. Moreover, the pyrolysis produces a porous non-graphitizing carbon material composed of graphene sheets and aluminosilicate nanoparticles, increasing the active surface area by around six times, leading to an outstanding electrochemical performance. 

A common challenge in portable food analyzer development, namely the necessity for additional validation and benchmarking in complex food matrices, mostly is neglected in µPED development as well. Only two studies benchmarked their devices against a well-established method [87,88]. Marín-Barroso et al. developed a µPED for the detection of gliadins in flour [88]. The device coupled screen-printed electrodes modified with carbon nanofibers to a paper-based immunoassay and used chronoamperometry for the detection, achieving an LOD of 3 mg∙kg^−1^, which is more than three times below the EU regulatory limit. The device was benchmarked against the official method for gliadin quantification in flour (ELISA assay) and the authors reported a correlation between the developed method and the ELISA with an R^2^ close to one. The selectivity of the sensor was determined by challenging it with albumin, casein, glutenin from wheat, β lactoglobulin, and folic acid. Only casein led to a 40% increase in signal, which was attributed to the cross-reactivity of the antibody against this target [89]. The complexity of the sample preparation, particularly for solid food samples, is another challenge common to several detection devices and it undermines the simplicity of the electrochemical detection systems. While three µPEDs were developed to detect contaminants in solid food, other studies focused on juices, milk, or alcoholic beverages [88,90,91]. Some of these studies mention LODs that are slightly above EU regulations. This includes a study on the detection of *Escherichia Coli* in cucumber and beef using a label-free impedimetric technique [90]. The reported LOD value was 1500 CFU·g^−1^ while EU regulation in ground beef stipulates at least two out of five samples must have a maximum of 50 CFU·g^−1^ (Commission Regulation (EC) No 2073/2005). 

**Figure 5:**
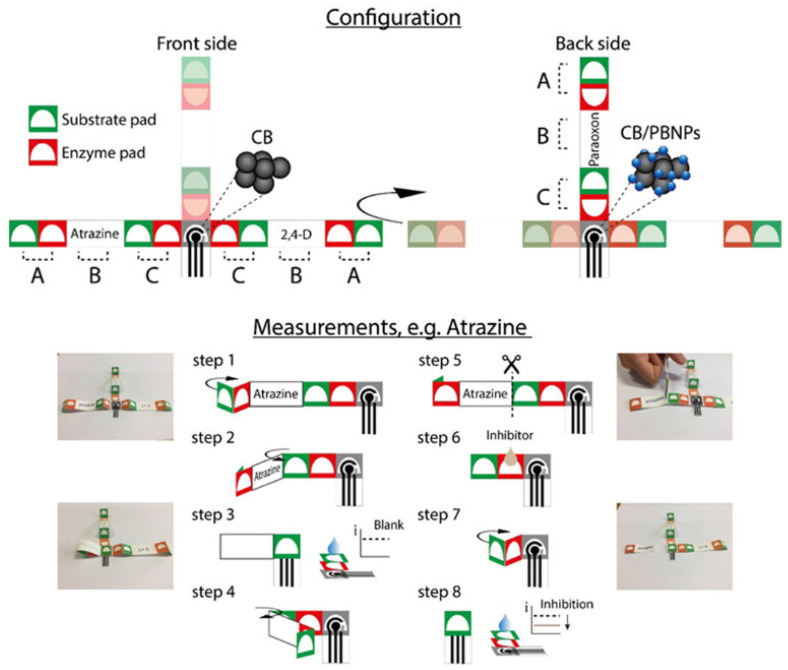
An example of a paper-based electrochemical food analyzer. The three-dimensional origami paper-based device was used for the detection of several classes of pesticides in river water based on the enzyme-inhibition assay. The multiplex analysis was achieved by folding and unfolding different parts of the paper-based origami structure. The measurement was performed with no need for adding reagents or sample preparation. Reproduced from [94] Biosensors and Bioelectronics, Copyright (2019), with permission from Elsevier.

The total assay time in the assessed µPED devices, like any other biosensing platform, depends on the assay type. The assays using conventional antibodies as recognition elements [88,90,92,93] never featured an analysis time below 30 min (not counting the extraction procedures), while the enzymatic assays can often be completed in under 15 min [94]. Finally, an interesting case was the development of origami-based µPEDs with pre-loaded reagents, allowing a simplified detection protocol (Figure 5) [94]. This device was used for multiplex pesticide detection (paraoxon, 2,4-dichlorophenoxyacetic acid, and atrazine) at ppb levels in river water samples. The detection was based on an enzyme-inhibition assay. The multiplex analysis was achieved by folding and unfolding different parts of the paper-based origami structure. The measurement was performed with no need for adding reagents or sample preparation. Another example of such devices has been developed for the detection of ethanol in beer [95]. A comprehensive protocol detailing the manufacturing of such a device has recently become available [96].

### 3.2. Microfluidic Chip-Based Electrochemical Food Analyzers

The integration of electrochemical biosensors within the microchannels of a chip-based microfluidic device is appealing due to the miniaturization capability of the electrochemical platform. Thus, it has been widely applied for the development of point-of-care and lab-on-a-chip devices for medical diagnostics. Moreover, an increasing trend toward the development of microfluidic solutions for food safety and environmental analysis has been observed [97,98]. Typically, electrochemical detection is incorporated within a microfluidic system by patterning electrodes on a flat glass or silicon substrate. Then a polymer-based layer of microchannels adheres to this substrate. Analogous to the microfluidic chips used for optical detection, the microchannels are fabricated mainly using polymers such as polydimethylsiloxane (PDMS), and Poly(2,5-dimethoxyaniline) (PDMA) due to the ease of fabrication and low cost [99]. 

The integration of microchannels and the use of flowing liquids can reduce the sample volume significantly, down to a few microliters, and increase the diffusion rate of the analyte molecules to the biorecognition element leading to a faster binding and biosensor response. Alternatively, the electroactive molecules can reach the electrode faster, increasing the electrochemical signal and improving the analytical performance of the device [100]. However, these advantages depend on the precise optimization of the flow rate and microchannel design. Apart from portability, other advantages of a chip-based electrochemical food analyzer include ease of integration and automation of multi-step procedures, high-throughput, and faster analysis. Therefore, these devices are attributed five stars for sensitivity, four stars for specificity, and three stars for rapid and robust consideration. However, they are not equipment-free and mostly require not only a potentiostat but also a pump, which provides them with only one star, exactly like the microfluidic chip-based optical devices. The need for additional equipment adds to the cost when compared to paper-based colorimetric and even electrochemical test strips, affecting the affordability of these devices (two stars) only slightly better than chip-based optical devices due to the lower price of a potentiostat compared to a miniaturized spectrometer. Thus, their overall score is 2.7. To circumvent the drawbacks, different high-throughput, automation, and multiplexing strategies have been explored when developing this class of devices (Table S6). 

Like the optical biosensing platforms previously discussed, the electrochemical biosensors also involve multi-step procedures, ranging from extracting the analyte from food samples to incubation with the eventual biorecognition elements and transport to the electrode. The microfluidic chip-based systems provide an easy way to integrate different modules and automate the overall procedure. Integration and automation of the multi-step biosensing process can effectively increase reproducibility and throughput while decreasing analysis time and cost. A very good example, showcasing the integration of different modules is a film-based integrated device developed by Park et al. for the detection of foodborne pathogens *Staphylococcus aureus* and *Escherichia coli* [101]. The device can perform multiple functions such as gene amplification, solution mixing, and electrochemical detection. The bacterial lysis was performed off-chip, then the extracted cell lysate was introduced to the device. After the amplification step, the target pathogen gene was detected by square wave voltammetry within 25 s, while gel electrophoresis detection requires about 30 min. 

**Figure 6:**
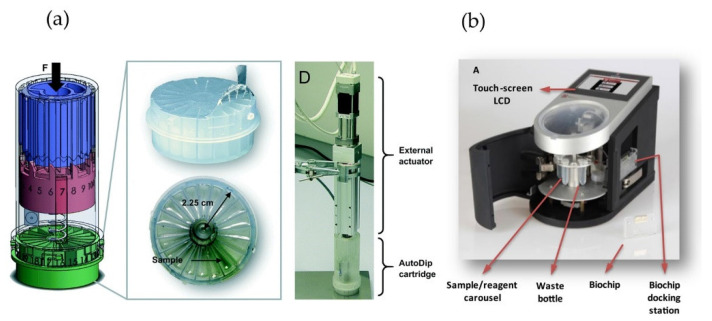
Examples of microfluidic chip-based electrochemical food analyzers. (**a**) AutoDip design and evaluation model. The reagent module is pre-filled with reagents, while an external vertical actuator is controlling the incubation and washing steps by consecutive dipping of the electrode into the reagents. Reproduced with permission from [102] lab on a chip, Copyright (2015), under Royal Society of Chemistry; (**b**) MiSense device designed for rapid and reliable detection of aflatoxin B1. The biochip attachment to the docking station creates a microfluidic channel on the top of the electrodes. Reproduced from [104] Talanta, Copyright (2016), with permission from Elsevier.

The fully automated electrochemical devices AutoDip and MiSense are the most advanced prototype examples, showing the applicability of microfluidic chip-based devices in the food safety field. The AutoDip is a fully integrated microfluidic platform with a user-friendly automated sampler, based on the ball-point pen mechanism (Figure 6a) [102]. This device consists of a disposable reagent module and an external actuator, controlling the incubation and washing steps by consecutive dipping of the electrode into the reagents. Therefore, the off-chip sample treatment is minimized with no need for microchannels, valves, or external pumps. As a showcase, the authors used the AutoDip device with a commercially available acetylcholinesterase biosensor (AC1.AChE, BVT Technologies, Czech Republic), to detect the pesticide chlorpyrifos with an LOD of 0.033 mg∙kg^−1^ in apple samples. Although this was below the MRL for this pesticide at the time of publication (0.5 mg∙kg^−1^), the MRL has recently been lowered to 0.01 mg∙kg^−1^ [103].

MiSense is an automated platform consisting of a biochip in an integrated microfluidic system (Figure 6b) [104]. The biochip is comprised of an array of 6 gold working electrodes with shared reference and counter electrodes on a silicon dioxide substrate. The device includes a biochip docking station, a pump, microfluidic tubing, reagent containers, and a waste bottle. It was used for aflatoxin B1 detection in wheat and fig samples with a limit of detection of 2 µg∙kg^−1^, which is the same as the MRL for aflatoxin B1 for cereals and below the MRL in fig samples [105]. The accuracy of the analysis was confirmed with a comparative study to the analogous ELISA test; correlation of the results resulted in a relationship with an R^2^ value of 0.96. The analysis time using MiSense was 25 min, shorter compared to 2–3 h for the ELISA test. Although MiSense is a portable platform providing fast and fully automated detection, the extraction procedure is not automated and involves the use of a solid-phase extraction cartridge, which adds to the time and cost of each test.

Several additional studies achieved a higher degree of automation by the creative design of the microfluidic platforms. One study developed a microfluidic chip-based device not only to detect the atrazine in orange juice but upon detection, to remove the contaminant from the juice, an interesting feature for industrial application [106]. The automated microfluidic platform is a hybrid polydimethylsiloxane–polyester chip, including a micromixer channel for efficient reagent mixing. After detection, atrazine was removed from the sample via anodic oxidation in the degradation chip. Other studies incorporated magnetic control functionality to the electrochemical microfluidic platform [107,108,109]. In these devices, the immunomagnetic separation minimized the non-specific response and reduced the time for immuno-reaction with active magnetic mixing [110]. 

Another key feature of electrochemical chip-based devices is the possibility of multiplexing measurements on disposable chips, resulting in cheaper and faster analysis. The simplest way to increase throughput is using multiple working electrodes for parallel electrochemical detection of a single analyte in multiple samples [111]. For example, in two studies, eight working electrodes were used for simultaneous measurement of *citrus tristeza* virus in citrus and *Salmonella typhimurium* in milk [112,113]. The cost of the disposable chip for citrus testing for eight simultaneous measurements was 1.99 US dollars per device, lower than the ELISA cost of 8.30 US dollars per microwell. The multiplexing of electrochemical measurements on microfluidic devices was achieved using dual-channel [114], separate sensing areas [115], or multiplex arrays [116] in different food matrices. From these studies, the multiplex device developed by Crew et al. for amperometric detection of six organophosphate pesticides is particularly interesting, since a neural network program was used for modeling the response [116]. This facilitated the interpretation of the analytical results, reducing the level of user training. The device was transported in a standard vehicle for field testing and powered from a car battery via the lighter socket. While the on-site testing was performed only with 4 different water samples, all the samples were identified negative for pesticide contamination with a 7.5% coefficient of variation between measurements.

One of the drawbacks of the microfluidic chip-based devices potentially limiting their on-site food safety application is the need for pumps and additional equipment. Several strategies have been used to circumvent this drawback. Lu et al. developed a dual-channel indium tin oxid (ITO)-microfluidics with capillary-driven PDMS channels for simultaneous electrochemical detection of two mycotoxins, fumonisin B1 and deoxynivalenol, in corn extracts [114]. The difference in the size of the inlet and outlet ports provided a capillary pressure difference, which drives the sample through the channel towards the outlet without a pumping unit. The LODs of 135 µg∙kg^−1^ and 175 µg∙kg^−1^ were achieved respectively for fumonisin B1 and deoxynivalenol, which are lower than the MRLs for these toxins [105]. Another good example is a low-cost, pump-free, capillary flow-driven microfluidic chip developed for the detection of *Salmonella* [117]. The flexible device is made of two polyethylene terephthalate (PET) layers, one containing the microchannels and the other substrate for the inkjet-printed electrodes and electrowetting valves. The last example is a self-pumping lab-on-a-chip device for automated detection of botulinum toxin in 15 min [118]. This PDMS-based integrated device consists of a mixer, an electrochemical sensing zone, and a capillary pump. The capillary pump provides steady flow, eliminating the need for externally powered pumps, simplifying the overall operation. 

Other notable studies of electrochemical chip-based microfluidic devices developed for food analysis include the use of novel biorecognition elements such as aptamers and molecularly imprinted polymers [119,120,121]. The microfluidic chip developed by Lin et al. was particularly innovative with a dual recognition platform using both molecularly imprinted polymers and aptamers, to detect carbofuran in vegetable and fruit samples [121]. Dual recognition improved the selectivity in complex food matrices. The PDMA microchannel included two functional areas. At the first functional area, the molecularly imprinted polymer adsorbs the carbofuran from the food extract, then the carbofuran is desorbed with the eluent buffer, flowing to the second functional area, to be captured by the aptamer on the electrode. Also, label-free impedimetric chips are of particular interest, since they provide a single-step measurement [122,123]. These devices are mainly reported for pathogen detection in food samples [124,125]. Considering that pathogens are much larger molecules than toxins, for the same number of bound molecules per surface area, they can increase the charge transfer resistance on the electrode much more. This means the system is more sensitive and less affected by impedimetric measurement drawbacks, such as nonspecific binding or environmental noise.

### 3.3. Smartphone-Based Electrochemical Food Analyzers

Smartphone-based biosensing devices have emerged as new bioanalytical tools in recent years, paving the road to citizen science owing to the smartphone’s wide accessibility, mass production, and capabilities for integration into lab-on-a-chip systems [126]. The electrochemical detection is of particular interest to be integrated into a smartphone-based device, being independent of the smartphone’s model variability. The smartphone in these examples was only used as the control unit of the potentiostat and as a user interface, displaying eventual commands and results. The improved accessibility (deliverable to end-users) and user-friendliness are strong features of this class of devices (three stars). However, their user-friendliness is graded lower than smartphone-based optical food analyzers, as the electrochemical training is more complicated than image analysis for non-experts. Their sensitivity and specificity are dictated by the electrochemical methods applied and were given five and four stars, respectively. Their affordability is lower (two stars) than their optical counterpart, the smartphone-based optical analyzers. This situation is due to the need for a potentiostat, which also reduces its performance as equipment-free, which is rated with two stars. Thus, their overall score is 3.1, still placing them in the top 3 most promising portable food analyzers, along with paper-based and smartphone-based optical food analyzers. While smartphone-based electrochemical devices have been developed for medical diagnostics and environmental analysis, there are very few examples of smartphone-based electrochemical biosensors for food safety testing [127,128,129,130,131].

**Figure 7:**
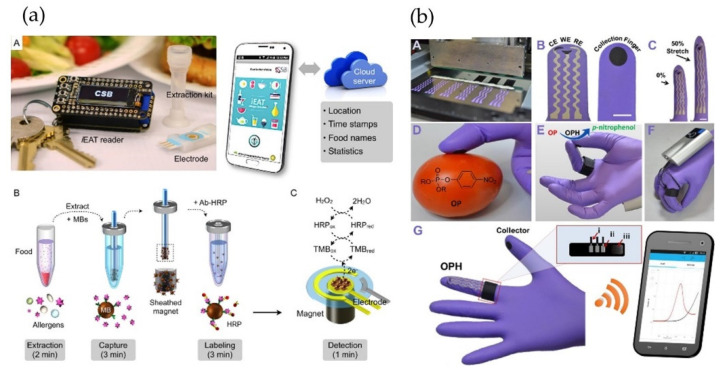
Examples of smartphone-based electrochemical food analyzers. (**a**) The iEAT platform. The keychain-sized detector is linked with a smartphone App. After extraction of the antigen from the sample, the detection is achieved by mixing HRP-labelled magnetic beads with the substrate (TMB, 3,3′,5,5′- tetramethylbenzidine) and moving it to the electrode. Reproduced with permission from [132] ACS Nano, Copyright (2017), American Chemical Society; (**b**) The lab-on-a-glove platform. The glove biosensor consists of a sampling finger and a sensing finger, containing the immobilized enzyme. Reproduced with permission from [133] ACS Sensors, Copyright (2019), American Chemical Society.

One of the two best examples of user-friendly and simple smartphone-based electrochemical food analyzers involves integrated exogenous antigen testing (iEAT) (Figure 7a) [132]. This device was used to detect food protein antigens with a detection limit of 0.1 mg·kg^−1^ in less than 10 min, at a cost of only 3 US dollars per test. The target protein antigens were gliadin in wheat, Ara h1 in peanut, Cor a1 in hazelnut, casein in milk, and ovalbumin in egg white. The integrated iEAT system consists of a disposable extraction kit, an electrode chip, a pocket-size potentiostat, which connects to a smartphone through a Bluetooth connection. The battery can be charged wirelessly with the smartphone. For data analysis, a smartphone App communicates with the iEAT device through Bluetooth and the data is uploaded to a cloud server. It can take photos of the users and analyze foods, set the detection channels and allergen types, display the measurement results, store the measurement time and location, and track the food intake history. The eventual measured presence of hazardous levels of antigens is displayed with a simple warning label, minimizing the need for user training. Although the system includes multi-step procedures, it is simple enough for non-expert users.

Another interesting device is the lab-on-a-glove developed by Wang’s group for the detection of organophosphorus compounds on fruit and vegetable surfaces (Figure 7b) [133]. The disposable glove biosensor consists of a sampling active area, printed on the thumb finger, while the enzyme-based biosensing detection area is printed on the index finger. The glove is coupled with a miniaturized potentiostat capable of real-time wireless data transmission to a smartphone. At the reported development stage, the results were qualitative and the user interface on the smartphone only showed the measured current plot. Recently, the same research group developed a quantitative glove biosensor for fentanyl (opioid) detection with an LOD of 10 µM [134]. Further development for a more user-friendly user interface would be needed before widespread application.

## 4. Portable Mass Spectrometry for Food Analysis

Conventional MS instrumentation is used primarily for confirmatory analysis in food safety applications. Nonetheless, recent studies report developments in the field of portable MS devices, which could be or are presented for applications in food analysis. We used the ASSURED criteria to assess the performance of the MS instrumentation in food safety screening applications. In terms of sensitivity and specificity, the MS instrumentation is an all-star (five stars), compared to all other screening assays described. The unequivocal identification is the main reason why MS is used as a confirmatory method according to the EU regulation 2002/657/EC [6]. The selection to monitor specific ions, characteristics for each substance, and the robust ion ratios, can guarantee the detection of specific food contaminants and differentiate between similar substances if they are not optical isomers. The rapidness is highly dependent on the ion source employed. In most portable MS applications for food safety monitoring, an ambient/direct ion source is employed, which significantly reduces the analysis time, however, the robustness of the ambient ion sources is not always guaranteed (three stars). Finally, the affordable, user-friendly, equipment-free, and deliverable to end-user characteristics are all weak (one star), contributing to an overall rating of 2.4. Mass spectrometers are significantly more expensive than the other screening assays described. They rely on relatively large and heavy instruments compared to screening assays and, with only a few exceptions, they require trained staff to operate the instrument and assess the result (Table 1).

Portable mass spectrometers are mainly applied in industry, environmental control, and forensics. Systems dedicated to food analysis are not commercially available yet. However, portable mass spectrometry (MS) is expected to find its way to on-site testing for food quality and safety parameters in the future. Historically, the first portable mass spectrometer was developed by John Hipple in 1942 for routine gas analysis [135]. Since then, a portable MS for gas analysis in the industry has evolved to the point of having a palm-sized MS system, weighing less than 2 kg, having a volume of fewer than 2 L, and operating with a 5 W battery, which is a significant improvement considering the conventional bulky benchtop instrumentation of up to 200 kg [136]. To transit from benchtop to portable MS instrumentation, all the components, including the mass analyzer, the ion source, the vacuum system, and the energy supply need to be miniaturized. Furthermore, other aspects such as sample clean-up, preparation, and separation need to be adjusted [137]. At the same time, these systems should be comparable in performance and capabilities with conventional benchtop instrumentation to be able to reach the relevant food safety detection levels and confirmation [138]. In practice, this means that compromises between performance, throughput rates, and cost need to be made [137]. 

Focusing on the mass analyzer, in a review published in 2016 by Snyder et al., over 30 miniaturized MS systems were discussed, which included different types of mass analyzers [139]. Among them, the quadrupole and ion trap mass analyzers are those used mostly in the field of food analysis. In contrast, miniaturization of the magnetic sector and time of flight (TOF) mass analyzers was quite a challenge, as their resolution depends on the length of the path the ions travel [140,141,142], and no application in the food safety field has been cited. For analyte ionization, atmospheric pressure ionization (API) methods, such as electrospray ionization (ESI), AP chemical ionization (APCI), and AP-Matrix-assisted laser desorption ionization (AP-MALDI) have been used in portable MS systems [137]. Moreover, with ambient or direct ionization (AI) sources, samples can be directly ionized with minimum or no sample preparation under ambient conditions, without any chromatographic separation. AIMS have been already applied in various food-related analytical methods [143] and can be easily coupled with miniaturized MS systems [144]. 

The ion trap mass analyzers reported for food safety applications are based on different generations of the Mini, a rectilinear ion trap analyzer developed at Purdue University (West Lafayette, IN, USA) equipped either with Low-Temperature Plasma (LTP), or Discontinuous Atmospheric Pressure Inlet (DAPI) ambient ionization. The Mini 10.5 is the first generation rectilinear ion trap analyzer that weighs 10 kg and can be operated on a 70 W battery [139]. This device was used by Huang et al. for the detection of melamine in whole milk, milk powder, and fish [145]. The authors chose heated air as a plasma carrier instead of helium to reduce the cost and to make it more portable without loss of sensitivity. The method is high-throughput; up to two samples can be analyzed per minute, and it has an LOD of 0.25 ppm for melamine in the milk sample, less than the EU regulatory limit of 1 ppm [146]. The Mini 10.5 was also used for direct analysis of two pesticides, diphenylamine, and thiabendazole in apples and oranges. Although this method did not provide sufficient quantification, it could differentiate between organic and non-organic fruits based on the detection of targeted pesticides [147]. Wiley et al. and Janfelt et al. used the mini 10.5 mass analyzer to detect food-safety-related compounds with a focus on several pesticides, including atrazine. Even though these two studies achieved adequate sensitivity compared to the benchtop Thermo LTQ linear ion trap MS, they did not perform real sample analysis, and the reported analysis suffered from low reproducibility [148,149]. 

The Mini 11 is the next-generation analyzer. At 5 kg, it weighs half as much as the Mini 10.5 and can be operated for two hours on a 35 W battery [139]. The Mini 11 was used in combination with ESI-MS, DESI-MS, and LTP-MS, to detect the origins of milk, fish, and coffee beans, respectively. The accuracy of the classification was high, enabling the characterization of adulterated food groups [150].

The next portable ion trap analyzer is the Mini 12 which can be operated with a 50 W battery [139]. Although the Mini 12 is heavier than the previous generations at 15 kg, it provides a high level of user-friendliness enabling non-expert users to perform the analysis. As demonstrated by Li et al., a peel of a conventionally grown orange was inserted in a paper spray cartridge, then the spray solvent was added and the ions were generated and detected [151]. In another study that involved the use of the Mini 12 by Pullian et al., direct leaf spray ionization was used to detect qualitatively the fungicide chlorothalonil in maple tree leaves even 5 days after its application [152]. Finally, in the last example of this ion trap analyzer, direct ionization slug-flow microextraction (SFME) nanoESI was applied to detect the plasticizer Bis(2-ethylhexyl)phthalate (DEHP) with an LOD of 5 ppm in spiked fruit punch, and of bisphenol A, a plastic monomer with hormone-like properties forbidden for use in infant formula bottles, with an LOD of 10 ppm in spiked milk sample [153]. A commercially available portable linear ion trap MS system has been developed by PurSpec Technologies, which weighs 20 kg and can be operated with a 100 W battery [154]. It was applied with SFME nanoESI for the detection of multiple fentanyl compounds, directly from beer, milk, or cola, with an LOD of 10 ppb, featuring low chemical noise [155]. 

Apart from ion trap analyzers, a single quadrupole MS has been implemented in portable food safety analysis as well. A good example is a technique developed by Zhang et. al. using a portable pyrolysis gas chromatography (GC) quadrupole MS with a total analysis time of only five minutes for detection of microplastics in seawater [156]. The high temperature selected for the pyrolysis (715 °C) ensured the full decomposition, ionization, and subsequent identification based on both generated ions and ion ratio. This technique was applied for in situ analysis of seawater, but it could be used, after further modification, for fish samples on fishing boats. A portable single quadrupole MS was also used in combination with a solid-phase microextraction (SPME) transmission mode followed by direct analysis in real-time (DART) ionization to identify pesticides in grape juice. In total, 3 pesticides were quantitatively analyzed with an LOD of 10 ppb, namely pyrimethanil, pyraclostrobin, and azoxystrobin, and 4 more were quantitatively analyzed with an LOD of 5 ppb, namely cyprodinil, metalaxyl, imazalil, and atrazine. The SPME-DART-portable single quadrupole MS approach was also applied to detect the origin of milk samples [157]. The complete analysis time was less than two minutes, which is significantly reduced compared to the standard benchtop SPME analysis with chromatographic separation [158,159]. Recently, Blokland et al. used a transportable single quadrupole MS system with different ionization methods, from which coated blade spray (CBS) showed the most promising results for the analysis of liquid food samples or extracts of solid foods [160].

Finally, a cutting-edge advancement in portable quadrupole MS development was the successful miniaturization of a triple quadrupole (QqQ) mass analyzer, being the gold standard in conventional food safety analysis by GC- or LC-MS. This development could pave the way for the on-site adaptation of standard methods used by routine food safety laboratories. The researchers demonstrated the application of the portable QqQ system using a standard LC column for thiabendazole detection in spiked apple pulp, achieving a total run time of six minutes and an LOD of 10 ppb, which is comparable to that of conventional benchtop instruments [161]. 

Other parts of the MS spectrometer that need to be adjusted for portable applications include the power supply and vacuum system. Nonetheless, significant improvements have been made in the field, as with the ionization part of the MS systems. For example, the power of the ion source, as Josha et al. demonstrated recently, could be supplied even by a USB interface plugged into a smartphone [162].

The ASSURED criteria are not directly/strictly applicable in MS analysis, since they are intended to assess screening assays. However, the use of MS, in terms of sensitivity and selectivity is superior compared to screening assays, thus on-field applications using portable MS could lead to improved food-safety monitoring. Most portable MS techniques described exploit simple and rapid direct or ambient ion sources without time-consuming chromatographic separation, which is already an improvement compared to confirmatory methods in routine laboratory analysis. From the techniques reviewed, most have focused initially on the detection of pesticides. Surprisingly, no techniques have been developed for the detection of natural toxins, even though they are a considerable risk for consumer’s health and a huge financial burden for the food industry [163]. This omission might be due to the fact that for some toxins a higher level of sensitivity is needed to reach the EU regulatory limit at μg∙kg^−1^ compared to some pesticides at mg∙kg^−1^, which is also considered the biggest challenge for portable MS development [105,164]. Also, the chemical structure of some of the pesticides favors high ionization efficiency, leading to lower detection limits. Improvements in instrumentation, miniaturization of the ion analyzer, pumping system, energy source, user-friendly interface, and development of AIMS source with minimal to no sample preparation highlight a path for further development.

As an intermediate solution to bridge the gap between portable biorecognition-based rapid screening and potential portable GC- or LC-MS for confirmatory analysis, direct MS without any chromatographic separation might be considered following the development of a dedicated LFA. In this context, the selectivity of the chromatographic separation is replaced by immuno-trapping on an LFA, where the antibodies isolate the analyte of interest. By subsequent dissociation of the analyte from the LFA, the retrieved solution can be directly analyzed by ESI or DART-MS. This approach offers possibilities as a confirmatory method maintaining the advantages of screening, such as rapid development and ease of use, with those of confirmation, providing unequivocal identification of the substance [165,166]. 

## 5. Conclusions

The increasing demand for reliable, rapid, and on-site food safety analysis is a motivating factor toward the development of fully integrated and automated portable food analyzers. Based on the ASSURED criteria, key trends in technological advancements have been identified that increase analysis sensitivity, combine complementary technologies, and involve full automation and integration of portable devices. Paper-based optical food analyzers are the simplest and most common platforms for on-site qualitative and semi-quantitative analysis, and there is a trend toward the development of affordable and portable readers to enhance their sensitivity and provide quantitative data. The development of hybrid devices, particularly combining LFA with electrochemical detection is the next leap toward meeting the ASSURED criteria by combining the complementary features of different platforms. Microfluidic chip-based electrochemical and optical food analyzers are good examples of integrated and automated platforms in portable food analysis. The design of fully automated and integrated devices, which include all necessary steps from sample preparation to detection, will bring them closer to the point-of-need analysis. Finally, although the portable MS analyzer is gaining momentum, it still lags in terms of affordability, robustness, and user-friendliness needed for point-of-need food safety analysis.

Despite many technological advancements, the new devices described in this review are still at the proof-of-concept stage. Although these devices usually have strong performance metrics within some of the ASSURED criteria, none of them meet all the requirements to be the ideal fit for purpose. Main limitations include lack of quantitation, automation, integration of sample preparation, and user-friendliness. Researchers should focus on both the ASSURED criteria and regulatory guidelines when developing such devices. While there are some notable examples involving the measurement of relevant analytes below regulatory limits in real samples, in most studies the platforms have not been validated in a real-life setting based on regulatory guidelines. This represents a critical gap for their implementation as food safety screening tools. Possible reasons for the lack of validation and benchmarking of portable food analyzers may relate to the required high sensitivity and reproducibility at analyte concentrations below the MRLs in complex food matrices. While real sample analysis is satisfactory in many cases, achieving a false negative rate equal to or lower than 5% in different food matrices, as required for validation studies, is a difficult task, requiring substantial additional research effort. Considering that validation and benchmarking studies play a crucial role in ensuring the applicability of new devices, stakeholders, granting bodies, and the food safety research community could more explicitly communicate the importance of and recognize the value of validation studies, and support and encourage technology developers to perform these studies. 

## Figures and Tables

**Table 1:** The Affordable, Sensitive, Specific, User-friendly, Rapid and Robust, Equipment-free, and Deliverable to end-users (ASSURED) criteria grading assigned in this comparative evaluation of different types of portable food analyzers. Five stars were given to the device with the highest performance in each criterion compared with other devices. As an example, the paper-based colorimetric device is the cheapest of the portable analyzers, so it was assigned five stars in the Affordability criteria. (star 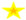).

Criteria	Affordable	Sensitive	Specific	User-Friendly	Rapid and Robust	Equipment-Free	Deliverable to End-user	Overall Score
Paper-based colorimetric	5	1	4	5	5	5	5	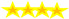 4.3
Smartphone-based optical	4	2	4	4	2	4	4	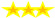 3.4
Smartphone-based electrochemical	2	5	4	3	3	2	3	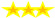 3.1
Microfluidic chip-based electrochemical	2	5	4	2	3	1	2	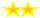 2.7
Microfluidic chip-based optical	2	4	4	2	3	1	2	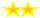 2.6
Paper-based electrochemical	3	2	4	3	3	2	2	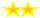 2.6
Raman/IR -based	2	1	5	3	4	2	2	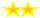 2.6
Portable MS as screening	1	5	5	1	3	1	1	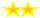 2.4

## Data Availability

The data presented in this study are available in [the supplementary material, spreadsheet Tables S1–S6].

## Supplementary Materials

The following are available online at https://www.mdpi.com/article/10.3390/foods10061399/s1, spreadsheet Table S1: paper-based optical food analyzers, spreadsheet Table S2: microfluidic chip-based optical food analyzers, spreadsheet Table S3: smartphone-based optical food analyzers, spreadsheet Table S4: Raman and IR-based portable food analyzers, spreadsheet Table S5: paper-based electrochemical food analyzers, spreadsheet Table S6: microfluidic chip-based electrochemical food analyzers.
